# Complete and Persistent Response to Immunotherapy in Highly Pretreated MSS TMB-High Pancreatic Adenocarcinoma: A Case Report and Literature Review

**DOI:** 10.3390/ijms27135722

**Published:** 2026-06-25

**Authors:** Chiara Carmen Miceli, Giuseppe Caropreso, Giovanni Pacifico, Erika Lara Valletta, Chiara Pisaniello, Maria Laura Sgura, Raffaella Carnevale, Fortunato Ciardiello, Ferdinando De Vita

**Affiliations:** Division of Medical Oncology, Department of Precision Medicine, University of Campania “Luigi Vanvitelli”, 80131 Naples, Italy; peppecarop96@gmail.com (G.C.); giovannipacifico94@gmail.com (G.P.); erikalara.valletta@studenti.unicampania.it (E.L.V.); chiara.pisaniello.97@gmail.com (C.P.); sguramarialaura@gmail.com (M.L.S.); raffaela.carnevale@studenti.unicampania.it (R.C.); fortunato.ciardiello@unicampania.it (F.C.); ferdinando.devita@unicampania.it (F.D.V.)

**Keywords:** pancreatic ductal adenocarcinoma, tumor mutational burden, microsatellite stability, immunotherapy, pembrolizumab, next-generation sequencing, genomic profiling, precision medicine

## Abstract

Despite advances in precision medicine, therapeutic options for pancreatic adenocarcinomas are limited, alongside a significant increase in incidence and mortality in recent years. We present the case of an exceptional response to immunotherapy in a heavily pre-treated pancreatic adenocarcinoma. The patient is a 73-year-old man that was diagnosed in 2017 with locally advanced pancreatic adenocarcinoma. He underwent different lines of chemotherapy and after exhausting standard treatment options, he practiced the FoundationOne^®^ CDx analysis (Foundation Medicine, Inc., Cambridge, MA, USA), that pointed out a High Tumor mutational burden that permitted our Oncology Center to request Pembrolizumab 200mg flat dose q 21 as an off-label therapy. The patient started the treatment in July 2021 and is still ongoing, having achieved a complete radiological response of hepatic metastases. Although immunotherapy is not part of the standard treatment paradigm for advanced pancreatic cancer, our case suggests that it may provide substantial and durable clinical benefit in a small molecularly selected subgroup of patients who have exhausted conventional therapeutic options, highlighting the critical role of comprehensive molecular profiling in identifying actionable treatment opportunities.

## 1. Introduction

Pancreatic cancer does not represent one of the most frequent neoplasia worldwide; it took up, indeed, the 12th place for incidence in the overall population, with nearly 510,992 new diagnoses in 2022. Nevertheless, it is one of the cancers with the poorest prognosis, responsible for almost the same number of deaths (467,409) in both sexes, ranking 6th in terms of mortality [1]. In Italy, pancreatic cancer ranks 7th in terms of incidence and 4th in terms of mortality, with a net survival rate of approximately 11% 5-years after diagnosis, continuing to represent a challenge both from a diagnostic and therapeutic point of view. Globally, its incidence is increasing by approximately 0.5–1% per year and is expected to become the second leading cause of cancer death by 2030 [2]. This contrasts with the steady increase in survival rates for most types of cancer: progress has been slow for pancreatic cancer, partly because more than half of cases are diagnosed at an advanced stage. Pancreatic ductal adenocarcinoma (PDAC) is the most common histotype, accounting for 90% of pancreatic neoplasms [3]. Approximately 50–55% of patients with pancreatic ductal adenocarcinoma present with metastatic disease at the time of diagnosis [1]. Although surgical resection is considered curative, the tumor recurrence rate after surgery remains high, with most patients experiencing a relapse of the disease. This has led to pancreatic ductal carcinoma being considered a systemic disease from the time of diagnosis, even in cases where the tumor is localized and apparently resectable. For this reason, even in patients with localized pancreatic adenocarcinoma, a multimodal treatment strategy, including surgery, chemotherapy and radiotherapy, may offer greater chances of survival [4,5]. In the metastatic setting, systemic chemotherapy with regimens such as mFOLFIRINOX, Gemcitabine + Nab-paclitaxel, PAXG and Nal-IRI + 5-fluorouracil is the standard treatment, with median survival often not exceeding one year [6,7,8,9,10].

Despite advances in precision oncology, treatment options for pancreatic ductal adenocarcinoma remain limited, and only a small proportion of patients benefit from biomarker-guided targeted therapies, such as PARP inhibitors in tumors with BRCA mutations after first line platinum-based chemotherapy [11]. Less than 1% of patients with MPC have high microsatellite instability (MSI-H) or mismatch repair deficiency (dMMR), often as part of Lynch syndrome. The KEYNOTE-158 study included 22 patients with MSI-H/dMMR MPC treated with pembrolizumab, with rather disappointing results [12,13]. Tumor mutational burden (TMB) has emerged as a potential predictive biomarker of response to immunotherapy in several types of cancer [14,15], although its role in pancreatic cancer remains unclear due to the rarity of cases with high TMB [16]. The ability to offer comprehensive mutational analysis at our center has enabled us to identify potentially actionable molecular alterations, allowing us to offer clinically relevant therapeutic opportunities, particularly in heavily pretreated patients who have exhausted standard treatment options.

We report here the case of a heavily pre-treated patient with metastatic pancreatic adenocarcinoma who achieved a complete and durable response to pembrolizumab after identification of a high tumor mutational burden by next-generation sequencing (FoundationOne^®^ CDx).

## 2. Case Report

In June 2021, a 73-year-old man came to our attention during an outpatient visit.

### 2.1. Clinical History

The patient is a 73-year-old man with no significant comorbidities except benign prostatic hypertrophy, for which he underwent TURP in November 2018. The patient reported occupational exposure to paints and dust, with no history of cigarette smoking; family history for cancer was unremarkable.

During the urological investigations carried out in preparation for TURP surgery, a chest CT scan with contrast medium was performed, which revealed, as an incidental finding, a densitometric alteration in the splenic hilum-parahilar area measuring approximately 5 cm, with a poorly defined cleavage plane with the tail of the pancreas, suggestive of a new formation in the pancreatic tail with organ infiltration. An upper and lower abdominal MRI with contrast medium was then performed, confirming the presence of a 4.5 cm expansive neoplasm in the tail of the pancreas, infiltrating the splenic hilum and left adrenal gland. He also measured Ca19.9 levels, which were 19,790 U/mL. He underwent EUS-guided FNAC, which diagnosed pancreatic adenocarcinoma. After discussion within the Multidisciplinary Team, the pancreatic neoplasm was considered locally advanced and inoperable, and therefore the patient was considered a candidate for systemic treatment with Gemcitabine + Nab-paclitaxel. From January to July 2019, the patient underwent systemic chemotherapy with gemcitabine and nab-paclitaxel for 6 cycles, resulting in a reduction in the size of the lesion. In August of the same year, he underwent splenopancreatectomy with left adrenalectomy with a diagnosis of moderately differentiated pancreatic ductal adenocarcinoma with mucinous component, pathological stage ypT3 ypN1 (1+/34), with minimal response to neoadjuvant therapy assessed according to Hartman criteria (grade 3). The patient was then referred for clinical and instrumental follow-up.

In December 2019, during follow-up checks, a recurrence of liver disease was detected, for which the patient began first-line chemotherapy with oxaliplatin and capecitabine from December 2019 to July 2020, achieving disease stability as the best response. The instrumental re-evaluation in August 2020 showed a new progression of liver disease. Therefore, from August 2020 to January 2021, he underwent second-line chemotherapy with capecitabine and irinotecan, again achieving disease stability as the best response. Subsequently, due to further progression of liver disease, from January 2021 to April 2021, he underwent third-line chemotherapy with gemcitabine rechallenge. Following further liver progression, the patient was considered eligible only for the best supportive care at the center where he was being treated.

### 2.2. Materials and Methods and Results

However, thanks to maintaining a good performance status, the patient continued to seek innovative treatment options, thus having the opportunity to undergo, in April 2021, a next-generation sequencing analysis tissue-based with the FoundationOne^®^ CDx platform on the tissue sample from the splenopancreatectomy surgery in August 2019. Genomic profiling analysis revealed a hypermutated signature, with a tumor mutational burden (TMB) of 35 mut/Mb, in the absence of microsatellite instability (microsatellite status: microsatellite stable, MSS). Typical PDAC driver alterations were identified, including KRAS G12D, along with inactivating mutations in tumor suppressor and DNA damage response (DDR) genes, such as TP53 R213* and a truncating mutation in MSH6 R922*, as well as a splice-site variant in CHEK2 (793-1G>A). Additional alterations were detected in genes involved in chromatin regulation (KMT2D/MLL2 G1235fs*95, SETD2 Q2030*), MAP2K4 M1V, and a splice-site variant in CASP8 (508-2A>G); PTEN G165R was reported as sub-clonal. No alterations were identified in BRCA1 or BRCA2. The results are summarized in Table 1.

**Figure 1:**
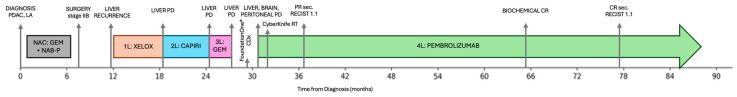
Clinical history of treatments and outcomes. Following a diagnosis of locally advanced PDAC, the patient underwent neoadjuvant chemotherapy and surgery, followed by several lines of systemic therapy for metastatic recurrence. After identification of a TMB-high/MSS molecular profile via FoundationOne CDx, treatment with pembrolizumab was initiated in July 2021, leading to a durable partial response according to RECIST 1.1 criteria, a subsequent complete biochemical response, and a complete radiological response that was still ongoing at the last follow-up. Abbreviations: PDAC: pancreatic ductal adenocarcinoma; LA: locally advanced; NAC: neoadjuvant chemotherapy; GEM: gemcitabine; NAB-P: nab-paclitaxel; 1L: first-line treatment; XELOX: Capecitabine plus Oxaliplatin; PD: progressive disease; 2L: second-line treatment; CAPIRI: Capecitabine plus Irinotecan; 3L: third-line treatment; RT: radiotherapy; PR: partial response; 4L: fourth-line treatment; RECIST: Response Evaluation Criteria in Solid Tumors; CR: complete response.

**Figure 2:**
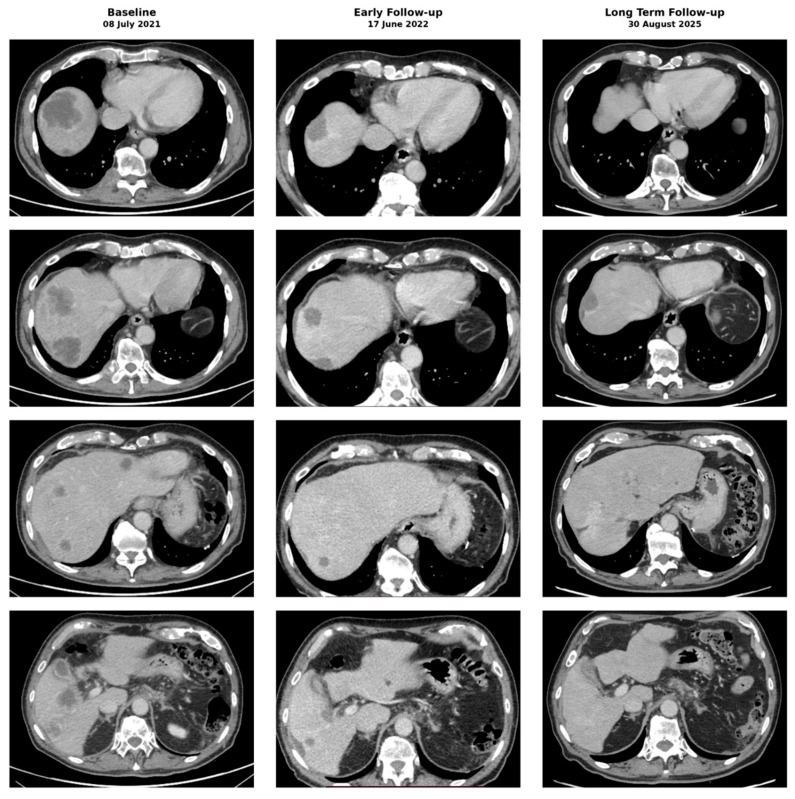
Radiological evolution of hepatic metastases during pembrolizumab treatment. Axial contrast-enhanced CT images obtained at baseline (8 July 2021), early follow-up (17 June 2022), and long-term follow-up (30 August 2025) are shown at corresponding anatomical levels. Baseline imaging shows multiple hepatic metastases. Early follow-up (17 June 2022) imaging demonstrates a partial response, with persistent disappearance of the target lesions at long-term follow-up, consistent with a sustained complete radiological response.

Given the TMB-high profile (≥10 mut/Mb) and the pan-tumor evidence supporting the use of anti–PD-1 therapy in tumors with high TMB [14,15], treatment with pembrolizumab was requested and initiated in an off-label setting. In June 2021, the patient was referred to our institution, where the off-label request for pembrolizumab was formally submitted. In July 2021, a repeat contrast-enhanced total-body CT scan revealed multiple hepatic metastatic lesions, the largest measuring 53 mm in segment VII and 64 mm in segment VIII, along with a paracaval pathologic lymph node (27 mm), a pericholecystic lymphadenopathy (24 mm), and an additional pathologic lymph node at the hepatoduodenal ligament (24 mm). The imaging also demonstrated malignant ascites with suspected peritoneal carcinomatosis, a destructive bone lesion involving the D10 vertebra (20 mm), and a 5 mm right periventricular brain metastasis. At the start of pembrolizumab treatment, the baseline Ca19.9 level was 18,950 U/mL and CEA was 446 ng/mL. After confirmation of the metastatic nature of the brain lesion by contrast-enhanced brain MRI, the patient underwent stereotactic radiotherapy using the CyberKnife system (Accuray Incorporated, Sunnyvale, CA, USA) in August 2021. Following approval of the off-label request, the patient started treatment with pembrolizumab 200 mg flat dose every 21 days in July 2021. At the first radiological reassessment performed in October 2021, who had shown disease stability, at the second radiological reassessment performed December 2021, a significant reduction in the size of the hepatic metastases was observed, with the lesion in segment VII measuring 37 mm and the lesion in segment VIII measuring 35 mm, corresponding to an approximate 39% decrease in the sum of target lesion diameters according to RECIST 1.1 criteria, consistent with a partial response. Previously enlarged lymph nodes showed a reduction with short-axis diameters < 10 mm. The previously described destructive lesion of the D10 vertebra was no longer detectable, while the brain metastasis remained stable. In December 2021 Ca19.9 was 812 U/mL and CEA was 7.22 ng/mL. In June 2024, a complete biochemical response was achieved, with normalization of tumor markers (Ca19.9: 29.2 U/mL; CEA: 2.07 ng/mL). Finally, at the contrast-enhanced total-body CT scan performed in August 2025, there was no evidence of hepatic, nodal, peritoneal and bone metastatic disease. The previously described metastatic lesions were no longer detectable. Brain MRI demonstrated findings consistent with post-radiotherapy radio-necrosis without evidence of active intracranial disease. Overall, the radiological assessment was consistent with a sustained complete response according to RECIST 1.1 criteria. At the most recent imaging assessment in January 2026, a sustained complete radiological response was confirmed, with normalization of tumor markers (Ca19.9: 28.5 U/mL; CEA: 2.48 ng/mL). The patient has been on pembrolizumab since July 2021, achieving an ongoing response duration of approximately 54 months. Treatment was exceptionally well tolerated, with no immune-related toxicities observed, and the patient maintained an excellent performance status and quality of life throughout therapy. The overall clinical course, including multimodal treatments, metastatic progression, molecular profiling, and treatment response, is summarized in the clinical timeline (Figure 1), while representative radiological findings documenting treatment response are shown in Figure 2.

## 3. Literature Review

### 3.1. Methods

We performed a PubMed search in March 2026 using combinations of the following keywords: pancreatic cancer, pancreatic adenocarcinoma, tumor mutational burden, TMB-high, hypermutated, microsatellite stable, MSS, pembrolizumab, nivolumab, and immune checkpoint inhibitors. We included case reports/series and observational cohorts reporting pancreatic ductal adenocarcinoma with documented TMB-high status treated with immune checkpoint blockade.

### 3.2. Results

A search on PubMed identified limited evidence on the use of immune checkpoint inhibitors in TMB-High pancreatic ductal carcinoma, mainly identifying case reports and case series, as well as a few retrospective and translational studies. Several case reports were identified that reported clinical benefit from the use of immune checkpoint inhibitors (Pembrolizumab, as in our case, or other PD-1 inhibitors) in patients with microsatellite stable (MSS)/mismatch repair–proficient (pMMR) pancreatic cancer and markedly elevated TMB [17,18,19,20,21,22,23,24,25,26,27]. The main clinical and molecular characteristics of published reports and observational studies are summarized in Table 2. Our research has highlighted that there are observational and translational studies reporting the existence of a rare subgroup of MSS/pMMR and TMB-high PDAC, suggesting that the presence of a high TMB may reflect a condition of ‘hypermutation’ even in the absence of microsatellite instability, characterized by better clinical outcomes in terms of survival when treated with immune checkpoint inhibitors, albeit extremely heterogeneous. Nevertheless, published case reports support the clinical relevance of comprehensive genomic profiling to identify patients with PDAC who are refractory to standard treatments but eligible for innovative therapies.

## 4. Discussion

It is well known that ductal adenocarcinoma of the pancreas is one of the neoplasms with the worst prognosis, just as it is well known that immune checkpoint inhibitors have not produced satisfactory results in either metastatic or localized settings [28,29,30,31,32,33].

Neoplastic cells are immersed in dense, fibrous connective tissue, the stroma, which contains fibroblasts and extracellular matrix proteins that create a physical barrier limiting the infiltration of immune cells. Furthermore, the presence of regulatory T cells (Tregs) and myeloid-derived suppressor cells (MDSCs) contribute to creating an immunosuppressive microenvironment, “cold”, characteristic of pancreatic adenocarcinomas and explaining the limited efficacy of immune checkpoint inhibitors [34,35,36,37,38,39,40,41].

Only 1–2% of patients with ductal adenocarcinoma of the pancreas have microsatellite instability or mismatch repair deficiency (MSI-H or dMMR), which often goes unrecognised as it is not routinely tested for in common clinical practice. Even in these cases, the use of immune checkpoint inhibitors has produced less than enthusiastic results, with an ORR of 18.2%, an OS of 4.0 months and a DOR of 13.4 months, far removed from what is seen in other solid tumor with microsatellite instability or mismatch repair deficiency (MSI-H or dMMR) [12,13]. The percentage of patients with ductal adenocarcinoma of the pancreas with high TMB represents approximately 1% to 4% of cases [16,22,23,25]. The data available in the literature on the actual efficacy of immune checkpoint inhibitors derive from case reports and small retrospective series, which suggests a potential sensitivity to immunotherapy in this patient subpopulation [16,17,19,24,42]. However, the evidence is still limited, especially in microsatellite stable (MSS) tumors [17,18,19,20,21,22,23,24,25,26,27].

We have described the case of an MSS patient with markedly elevated TMB (35 mutations/Mb) who achieved a durable complete response to pembrolizumab, which helps to reinforce the hypothesis that there is a subgroup of patients with unique biological characteristics who may still benefit from immune checkpoint inhibition.

An interesting element of our case is the coexistence of microsatellite stability (MSS) in the presence of an MSH6 R922* nonsense mutation, which causes a stop signal with the production of a truncated protein, considered pathogenic for Lynch syndrome [43,44]. Typically, MSH6 alterations are associated with a dMMR phenotype, but several mechanisms could explain the observed discordance. First, monoallelic or incomplete inactivation of the gene could lead to an increase in mutational burden without producing a detectable MSI-H phenotype. Secondly, intratumoral heterogeneity or subclonality of the MMR defect could lead to an underestimation of MSI status while contributing to genomic instability. These mechanisms may increase the overall mutational burden without generating a detectable MSI-H phenotype [45,46,47,48].

It is important to emphasise that TMB and MSI represent distinct biological parameters: while microsatellite instability is an indicator of a defect in the mismatch repair system resulting in replication errors in repetitive DNA sequences, TMB measures the overall mutational burden. Therefore, tumors with high TMB can be observed in the absence of microsatellite instability [49]. Thus, although MSI and TMB represent distinct but partially overlapping biological phenomena, MSS/TMB-high phenotypes have also been described in the literature in other solid tumors and may identify biologically distinct subgroups with greater immunogenicity [23,49,50,51,52,53].

In our case, mutations in genes involved in the response to DNA damage, such as CHEK2 and TP53, together with alterations in chromatin regulation genes (KMT2D/MLL2, SETD2), may have contributed to genomic instability and the generation of tumor neoantigens, justifying the presence of high TMB and reinforcing the hypothesis that our patient has a hypermutated and genomically unstable phenotype. Overall, these alterations may have created a biological context conducive to the immunotherapeutic response observed [54,55,56,57,58,59,60].

The role of TMB as an ‘agnostic’ biomarker in solid tumors was evaluated in the KEYNOTE-158 study, which led to the FDA’s approval of pembrolizumab for solid tumors with high TMB (≥10 mut/Mb) after progression on standard therapies [14]. However, the use of pembrolizumab in this setting is not possible in Italy: the possibility of receiving the drug off label at our center, which has extensive experience in the treatment of pancreatic cancer, allowed us to achieve durable disease control.

However, the predictive value of TMB in PDAC remains unclear due to the rarity of hypermutated cases. The present case contributes to the growing evidence suggesting that TMB-high PDAC may represent a clinically relevant subgroup that is potentially treatable with immunotherapy, even in the absence of MSI.

This case also highlights the importance of integrating molecular profiling into clinical practice. Genomic analysis performed after standard treatment options had been exhausted identified an actionable biomarker that substantially altered the patient’s treatment journey. These data support the use of next-generation sequencing in advanced PDAC, especially in patients with good performance status who may benefit from personalized approaches.

Another noteworthy feature is the extraordinary duration and depth of the response. Complete responses observed after 4 years of treatment and sustained over time are exceptional in metastatic pancreatic cancer, particularly in heavily pre-treated patients.

The patient described can be considered an exceptional responder to immunotherapy: the combination of complete radiological response, normalization of tumor markers and a response duration of more than four years represents an extremely rare outcome in this disease.

## 5. Conclusions

In conclusion, this case demonstrates that a subgroup of patients with metastatic pancreatic adenocarcinoma characterized by high tumor mutational burden can derive significant and lasting clinical benefit from immune checkpoint inhibition, even in the absence of microsatellite instability. The coexistence of MSS status with mismatch repair gene alterations highlights the biological complexity of hypermutated tumors and emphasizes the importance of comprehensive molecular characterisation integrating NGS and immunohistochemistry, to open up “agnostic” therapeutic opportunities in settings where standard therapeutic options are lacking. Prospective studies are needed to better define the predictive role of TMB and other genomic biomarkers in pancreatic cancer and to optimize the selection of patients eligible for immunotherapy.

## Figures and Tables

**Table 1:** Genomic alterations identified by FoundationOne^®^ CDx comprehensive genomic profiling.

Gene	Genomic Alteration	Variant Type	Variant Allele Frequency (VAF)	Functional/Pathway Implication
KRAS	G12D	Missense (activating)	22.4%	Oncogenic driver; MAPK signaling activation
TP53	R213*	Nonsense (loss of function)	34.3%	Tumor suppressor loss; genomic instability
MSH6	R922*	Nonsense (truncating)	33.4%	DNA mismatch repair pathway alteration
CHEK2	793-1G>A	Splice-site	43.3%	DNA damage response impairment
KMT2D (MLL2)	G1235fs*95	Frameshift	26.4%	Chromatin remodeling dysregulation
SETD2	Q2030*	Nonsense	24.6%	Histone methylation/epigenetic regulation
MAP2K4	M1V	Missense (likely LOF)	20.3%	Stress signaling/tumor suppressor pathway
CASP8	508-2A>G	Splice-site	6.6%	Apoptosis signaling alteration
PTEN	G165R (subclonal)	Missense	0.91%	PI3K/AKT pathway activation (subclonal)

Data derived from FoundationOne^®^ CDx assay. Abbreviations: LOF: loss of function; MAPK: mitogen-activated protein kinase; PI3K: phosphoinositide 3-kinase; AKT: protein kinase B; *: stop codon.

**Table 2:** Published case reports and cohort studies of tumor mutational burden–high pancreatic cancer treated with immune checkpoint inhibitors.

Study	Tumor Type/Setting	MSI/MMR Status	TMB (mut/Mb) and Method	Immune Checkpoint Therapy	Best Response	Duration of Response	Relevant Molecular Features/Notes
Zhu et al., 2022 [17]	Advanced pancreatic cancer	MSS/pMMR	11.7 (tissue NGS)	Pembrolizumab-based combination	Partial response	PFS ~9 months	Demonstrates clinical benefit despite MSS status
Zhang et al., 2022 [18]	Metastatic PDAC	MSS	49.9 (tissue NGS)	Sintilimab (anti–PD-1)	Clinical response	Not reported	Very high TMB; hypermutated phenotype
Chen et al., 2019 [20]	Advanced pancreatic cancer	pMMR	High TMB (ctDNA)	Anti-PD-1 + anti-angiogenic therapy	Partial response	PFS ~7 months	Blood-based TMB used as biomarker
Lundy et al., 2022 [26]	Metastatic pancreatic cancer	MSS	Not specified	Pembrolizumab + olaparib	Complete response	NR	Combination therapy; MSS responder
Dai et al., 2023 [24]	Metastatic PDAC	Not specified	Very high TMB (ctDNA)	Pembrolizumab monotherapy	Partial response	PFS ~2 months	First report using ctDNA-guided pembrolizumab
Li et al., 2024 [19]	Metastatic PDAC	MSS	PD-L1 CPS 75; PD-L1 TPS 70%; MSS	Pembrolizumab	Partial response	PFS ~13 months	Highlights role of PD-L1 as biomarkers
Quintanilha et al., 2023 [21]	Real-world PDAC cohort	MSI-H and MSS	TMB stratified	ICIs monotherapy	NA	NA	Observational evidence supporting predictive value of TMB
Kai et al., 2026 [22]	Unresectable/recurrent pancreatic cancer	MSI-H and MSS	10–53.5 (tissue NGS)	Pembrolizumab	ORR 33.3%, DCR 66.7%	PFS 90 days (MSS/TMB-high); PFS 227 days (MSI-H/TMB-high)	ICI showed limited activity in MSS/TMB-high tumors

Data extracted from published case reports and observational studies evaluating immune checkpoint inhibitors in pancreatic cancer with elevated tumor mutational burden. Abbreviations: PDAC: pancreatic ductal adenocarcinoma; MSS: microsatellite stable; MSI-H: microsatellite instability–high; MMR: mismatch repair; pMMR: proficient mismatch repair; TMB: tumor mutational burden; NGS: next-generation sequencing; ctDNA, circulating tumor DNA; ICI: immune checkpoint inhibitor; ORR: objective response rate; DCR: disease control rate; PFS: progression-free survival; OS: overall survival; NR: not reported; NA: not applicable.

## Data Availability

The original contributions presented in this study are included in the article. Further inquiries can be directed to the corresponding author.
